# Compatibility between Entomopathogenic Fungi and Egg Parasitoids (Trichogrammatidae): A Laboratory Study for Their Combined Use to Control *Duponchelia fovealis*

**DOI:** 10.3390/insects11090630

**Published:** 2020-09-14

**Authors:** Emily Silva Araujo, Alex S. Poltronieri, Carolina G. Poitevin, José Manuel Mirás-Avalos, Maria Aparecida Cassilha Zawadneak, Ida Chapaval Pimentel

**Affiliations:** 1 Laboratory of Entomology Professor Ângelo Moreira da Costa Lima, Department of Basic Pathology, Federal University of Paraná, Av. Cel. Francisco H. dos Santos, s/n, Curitiba 81531-980, Paraná, Brazil; mazawa@ufpr.br; 2 Departamento de Fitotecnia, Centro de Ciências Agrarias, Federal University of Santa Catarina, Rodovia Admar Gonzaga, 1346, Florianópolis 88034-000, Santa Catarina, Brazil; alexpoltronieri@gmail.com; 3 Laboratory of Microbiology and Molecular Biology (LabMicro), Department of Basic Pathology, Federal University of Paraná, Av. Cel. Francisco H. dos Santos, s/n, Curitiba 81531-980, Paraná, Brazil; carol.poitevin@gmail.com (C.G.P.); ida@ufpr.br (I.C.P.); 4 Unidad de Suelos y Riegos (asociada a EEAD-CSIC), Centro de Investigación y Tecnología Agroalimentaria de Aragón (CITA), Av. Montañana, 930, 50059 Zaragoza, Spain

**Keywords:** biological control, European pepper moth, Hypocreales, integrated pest management (IPM), *Trichogramma atopovirilia*, *Trichogramma pretiosum*

## Abstract

**Simple Summary:**

The European pepper moth is an important pest of many crops; however, some countries, such as Brazil, do not have insecticides registered for combating this pest. In particular, the egg stage of the biological cycle of this moth is the most difficult life stage to control. In this sense, biological control agents, including egg parasitoids and entomopathogenic fungi, can be an alternative to pesticides. In this study, laboratory tests were conducted to evaluate the susceptibility of two species of egg parasitoids (Trichogrammatidae family) to entomopathogenic fungal strains (including two commercial bioinsecticides). These fungal strains were applied on eggs of the European pepper moth before and after parasitism by the parasitoids. Overall, the entomopathogens reduced the parasitism rate, adult emergence, and longevity of adult parasitoids by less than 30%. The results obtained constitute the first step in designing effective pest control strategies. Future research should investigate the sub-lethal effects of the fungal strains on the parasitoids in the field.

**Abstract:**

The European pepper moth, *Duponchelia fovealis* (Lepidoptera: Crambidae), is a key pest in strawberry production. Entomopathogenic fungi (EF) and parasitoids of the Trichogrammatidae family are effective biological control agents of this pest with the potential to be used jointly for improved efficacy. This study aims to evaluate the susceptibility of *Trichogramma atopovirilia* and *Trichogramma pretiosum* to two *Beauveria bassiana* strains (B2 and B3) and two commercial bioinsecticides (Bovemax^®^ and Methamax^®^) by applying them to *D. fovealis* eggs in pre- and post-parasitism periods. Pre-parasitism application of B2 and B3 did not affect the percentage of *D. fovealis* eggs parasitized by either *Trichogramma* species, except in the case of *T. atopovirilia* when eggs were sprayed with B3 at 1.5 × 10^5^ conidia mL^−1^ (16.7% less than the control). In contrast, eggs sprayed with 1.5 × 10^8^ conidia mL^−1^ of the commercial bioinsecticides were not parasitized by any *Trichogramma* species. Overall, the EF tested reduced the parasitism rate, adult emergence, and longevity of *Trichogramma* adults by less than 30% in all cases. The adverse effects of the *B. bassiana* strains and commercial products on the biological traits of both *Trichogramma* species were minimal, meaning that these agents can be used jointly in *D. fovealis* control strategies.

## 1. Introduction

In Brazil, strawberry (*Fragaria x ananassa*) cultivation is of particular socioeconomic importance because of its prevalence in small rural properties, providing farmers with additional income [1]. Damage due to disease and pests over the growing season can severely limit yields. The European pepper moth, *Duponchelia fovealis* (Lepidoptera: Crambidae), which is native to the Mediterranean region, has become a major pest in strawberry fields in Brazil [2,3]. Its activity in strawberry cultivation has been previously noted in Portugal, Italy, France, and Turkey [4,5,6]. *Duponchelia fovealis* larvae feed on leaves and flowers, resulting in physical damage that can create entry points for pathogenic microorganisms and increase the risk of crop losses [3]. No authorized chemical pesticides are currently available in Brazil to control this exotic pest [7].

In this context, biological agents are important tools for controlling *D. fovealis* populations in strawberry plantations. Hymenoptera parasitoids represent one of the most important functional groups in insect pest regulation and are used in many crop systems worldwide [8]. Species of the Trichogrammatidae family are parasitoids that develop in the eggs of a wide range of insect species, making them one of the leading biological control agents employed worldwide. Indeed, they are involved in pest control programs for over 200 insect species [8]. In particular, members of the Trichogrammatidae family have considerable potential as cost-effective control agents against Lepidoptera pests due to their ability to be easily mass-produced [8,9,10,11,12]. However, the use of non-selective pesticides in agricultural production systems limits the successful implementation of biological control programs that use Trichogrammatidae species due to the sensitivity of these organisms [11]. Moreover, pesticide application can negatively affect beneficial arthropods, cause toxicity to humans, and may contaminate the final crop products, thus becoming especially relevant for those that are consumed in fresh, such as fruits and vegetables, including strawberries [13]. Effectively integrating different biological control agents could, therefore, minimize the use of aggressive chemicals.

Entomopathogenic fungi (EF) are a potential component of integrated pest management (IPM) programs for strawberry cultivation [14]. While some studies have reported laboratory infections of non-target organisms [15,16,17], EF are less effective under field conditions [18], and generally do not harm ecosystem service providers such as pollinators, earthworms, predatory arthropods, and parasitoids, when at low concentrations. However, given the potential of EF to have negative side effects on biocontrol agents [19], preliminary studies are necessary to evaluate the compatibility of EF with other beneficial organisms. This highlights the need for research on the selectivity and effectiveness of commercial microbial insecticides against *D. fovealis* since this may uncover opportunities to implement sustainable strawberry pest management strategies. In 2010, during the collection of *D. fovealis* individuals in Paraná state, Brazil, researchers observed that larvae and pupae of this species were infected by *Beauveria bassiana* ((Balsamo-Crivelli) Vuillemin) (Hypocreales: Cordycipitaceae) [3]. Subsequent spray applications of two *B. bassiana* strains that were isolated from Coleoptera species present in strawberry crops caused high mortality among *D. fovealis* larvae (>60%) [20]. Recent research on *B. bassiana* infections of *D. fovealis* has shown that although the fungus effectively controlled larvae and pupae, it did not affect the eggs [21]. Indeed, eggs are considered to be the most difficult insect life stage to kill because of their protective structure, which provides a barrier against penetration by insecticides and pathogens [22,23,24].

In the field, *D. fovealis* deposits its eggs on the abaxial side of strawberry leaves [2], reducing the effectiveness of chemical applications. This appears to suggest that the combined use of EF and egg parasitoids could increase pest mortality. However, there is a paucity of information available about the use of egg parasitoids for controlling *D. fovealis*. Trials involving *Trichogramma* species on *D. fovealis* eggs indicated that *Trichogramma galloi* Zucchi and *Trichogramma pretiosum* Riley (Hymenoptera: Trichogrammatidae) are promising at controlling the pest’s eggs [9]. Studies have also determined the ideal number of such biological agents to be released in strawberry fields [25]. Other species from the Trichogrammatidae family have been reported as effective biological agents against *D. fovealis*, including *Trichogramma exiguum* Pinto and Platner, *Trichogramma atopovirilia* Oatman and Platner [10], *Trichogramma cacoeciae* Marchal, and *Trichogramma brassicae* Bezdenko [26].

Combining biological control agents like egg parasitoids and EF may result in more effective pest control [16,19,24]. For instance, the selectivity of *B. bassiana* and *Metarhizium anisopliae* (Metschnikoff) Sorokin to *T. pretiosum* was assessed previously, and the results suggested that the simultaneous use of both EF with this parasitoid is possible [27]. Even though *M. anisopliae* decreased emergence and increased mortality among the parasitoids, there were no significant changes in the number of parasitized eggs and sex ratio when *M. anisopliae* was applied pre-parasitism [27]. However, the risk of using *B. bassiana* has been shown to vary depending on the fungal strain, insect host, and parasitoid being evaluated [22]. The interaction between *T. pretiosum* and the two strains of *B. bassiana* used against *Ephestia kuniella* Zeller (Lepidoptera: Pyralidae) was considered innocuous because the parasitism rate was reduced by less than 30% when these fungal strains were applied either pre- or post-parasitism [22]. Given these results, further studies are needed to determine how to combine different biological control agents without impairing their effectiveness while developing strategies that may improve their efficacy [16,17].

This study aims to assess the susceptibility of *T. pretiosum* and *T. atopovirilia* to two *B. bassiana* strains and two commercial bioinsecticides applied to eggs of *D. fovealis*, either pre- or post-parasitism, in non-choice tests. In addition, a free-choice bioassay was conducted. The resulting information will be useful for designing future biological control strategies in IPM programs against *D. fovealis*.

## 2. Materials and Methods

### 2.1. Duponchelia fovealis Rearing

Specimens of *D. fovealis* were obtained from a laboratory colony (Universidade Federal do Paraná, Curitiba, Paraná, Brazil) established from locally collected insects and reared at 25 ± 2 °C, 70 ± 10% RH, and 14 L:10 D photoperiod [28]. Adults were kept in plastic cages (15 × 15 × 12.5 cm) and fed on a nutritional solution consisting of 0.5 g nipagin, 0.5 g sorbic acid, 30.0 g sugar, 10 mL honey, 170 mL beer, and 500 mL distilled water. The walls of these cages were wrapped with a paper towel for egg deposition. Strips of paper towels containing eggs were fixed in sterile, vented, plastic containers (7 × 4 × 4.5 cm). Larvae were fed on an artificial diet consisting of seeds of *Phaseolus vulgaris* L. “carioca” cultivar (65 g), wheat germ (50 g), textured soy protein (25 g), casein (25 g), beer yeast (31 g), bacteriological grade agar (20 g), ascorbic acid (3 g), sorbic acid (1.5 g), methylparaben-nipagin (2.5 g), tetracycline (0.14 g), 30% formaldehyde (3 mL), Vanderzant vitamin mixture (8 mL), and distilled water (1000 mL), to which V8^®^ vegetable juice (50 mL) was added as a phagostimulant [28]. Pupae were transferred to sterile Petri dishes (15 × 2 cm) with moist filter paper until adult emergence.

### 2.2. Trichogramma Rearing

To obtain a sufficient population of *T. pretiosum* and *T. atopovirilia*, these parasitoids were reared on *Mythimma sequax* Franclemont (Lepidoptera: Noctuidae) eggs. Newly hatched *M. sequax* larvae were kept in plastic containers (5 × 4 cm) and fed on an artificial diet until they reached the 4th instar [29]. They were then individually placed in glass tubes (2 × 8 cm), where they were kept until they reached the pupae stage. Adults were kept in cages (45 × 30 × 22 cm) and fed on honey (10%) and water. Strips of paper were provided for oviposition, and the eggs were used to maintain the *Trichogramma* populations. Rearing was conducted under controlled conditions (20 ± 1 °C, 70 ± 10% RH, and 12 L:12 D photoperiod). Populations of *T. pretiosum* and *T. atopovirilia* were kept in glass tubes (1 × 10 cm) under the same controlled conditions as those described for *M. sequax*. At 48 h intervals, approximately 100 *M. sequax* eggs were offered for parasitism. Adult parasitoids were fed diets of pure honey.

### 2.3. Entomopathogenic Fungal Strains and Commercial Bioinsecticides

*Beauveria bassiana* B2 (Genbank: KU751847) and B3 (Genbank: KU751848) strains were selected because they have been shown to cause high mortality rates in *D. fovealis* (>60%) in previous studies [20]. Monospore cultures of each isolate were inoculated into Petri dishes containing Niger Seed Agar (NSA) and incubated for 14 days (28 ± 2 °C) [30]. The mycelia and conidia were subsequently scraped from the Petri dishes and transferred to vials (20 mL) containing 0.85% saline solution (NaCl containing 0.01% Tween 80). The vials were agitated on a vortex mixer, and the resulting suspensions were filtered to remove mycelial fragments. The concentrations of the resulting suspensions were determined by hemocytometer counts and were adjusted to 1.5 × 10^5^ and 1.5 × 10^8^ conidia mL^−1^. The viability of the isolates and bioinsecticides was verified by spraying 100 μL of a suspension containing 10^4^ conidia mL^−1^ onto Petri dishes containing Sabouraud Agar. The dishes were maintained at 25 °C and 100% RH for 16 h. Viability was determined by viewing 200 conidia under a microscope (400× magnification). Conidia that had produced a germ tube were considered viable. This procedure was used for all isolates, and viability was >90% in every case.

In addition, the following two commercial bioinsecticides available as emulsifiable concentrates (EC) were used as positive controls in the bioassays: Bovemax^®^EC (*Beauveria bassiana* Strain CG 716, containing 1.5 × 10^9^ viable conidia per mL of commercial product) and Methamax^®^EC (*Metarhizium anisopliae* Strain IBCB 348, containing 2.5 × 10^9^ viable spores per mL of commercial product). These bioinsecticides were purchased from Novozymes BioAg (Brazil) and were diluted to obtain test concentrations of 1.5 × 10^5^ and 1.5 × 10^8^ conidia mL^−1^. The highest of these two concentrations corresponds to the recommended dose for field applications. Throughout this paper, Bovemax^®^EC and Methamax^®^EC will be referred to as Bb CG716 and Ma IBCB348, respectively.

### 2.4. Trichogramma atopovirilia and Trichogramma pretiosum Parasitism in Free-Choice Test

Paper strips with 20 eggs (24 h) of *D. fovealis* (from the population reared as described in Section 2.1) were sprayed with 0.5 mL of a suspension containing either 1.5 × 10^5^ or 1.5 × 10^8^ conidia mL^−1^ of each *B. bassiana* strain, commercial bioinsecticide, or the control (sterilized distilled water + 0.01% Tween 80). Spraying was performed using an airbrush (Pneumatic Sagyma^®^ SW775, São Paulo, SP, Brazil) coupled to a pump at constant pressure (1.2 kgf cm^−2^). After being sprayed, paper strips were left to dry in a flow chamber for 1 h. A single paper strip sprayed with a fungal treatment was then placed into a sterilized glass tube (2.5 × 8.0 cm), along with a non-sprayed control and a *T. atopovirilia* or *T. pretiosum* female (24 h old) from the population reared as described in Section 2.2. The tubes were closed and kept under controlled conditions (25 ± 2 °C, 70 ± 10% RH, and 12 L:12 D photoperiod).

After 24 h, the parasitoids were collected and isolated in glass tubes. After their death, they were transferred into sterilized Eppendorf tubes containing moistened cotton and kept at 28 ± 2 °C for 14 days to verify the occurrence of fungal extrusion. The paper strips with eggs were placed into separate glass tubes (5 × 1 cm) and kept under the same controlled conditions. The percentage of parasitism by each *Trichogramma* species was assessed using a free-choice test based on the numbers of parasitized eggs on the control and treated paper strips. Specifically, this test compared each *B. bassiana* strain (B2 and B3) or bioinsecticide (Bb CG716 and Ma IBCB348) with its corresponding control. Fifteen replicates per treatment combination were performed. Each replicate consisted of a tube with two paper strips (one treated and the other non-treated) with 20 *D. fovealis* eggs each and a female parasitoid.

### 2.5. Spraying of Entomopathogenic Fungi Pre- and Post-Parasitism (No-Choice Tests)

The pre-parasitism bioassay was performed using 0.5 mL suspensions containing 1.5 × 10^8^ conidia mL^−1^ of each *B. bassiana* strain or commercial bioinsecticide, or the control (sterilized distilled water + 0.01% Tween 80) sprayed on unparasitized eggs of *D. fovealis* (ca. 24 h old, from the population reared as described in Section 2.1) fixed on paper strips. Spraying was conducted with the same equipment as in the bioassay described in Section 2.4. After being sprayed, paper strips were left to dry in a flow chamber for 1 h. Then, 15 paper strips (containing 20 *D. fovealis* eggs each) for each fungal strain, commercial bioinsecticide, and control groups were placed into separate sterilized glass tubes (2.5 × 8.0 cm) with a *T. atopovirilia* or a *T. pretiosum* female (24 h old) from the population reared as described in Section 2.2. The tubes were closed and kept for 24 h under controlled conditions (25 ± 2 °C, 70 ± 10% RH, and 12 L:12 D photoperiod). The parasitoids were transferred into glass tubes and, after their death, they were isolated in sterilized Eppendorf tubes and kept at 28 ± 2 °C for 14 days to verify the occurrence of fungal extrusion. Mortality due to the fungus was confirmed by observing the insect under a stereomicroscope at 20× or 40× magnification [31].

In the post-parasitism bioassay, a female of *T. atopovirilia* or *T. pretiosum* (24 h old) was confined with a paper strip containing 20 *D. fovealis* eggs in a glass vial for 24 h. After this period, the parasitoids were removed, and the paper strips were sprayed in the same manner as in the former bioassay and kept for 1 h in a flow chamber. Then, the paper strips were isolated in glass tubes and kept under the same controlled conditions as previously described. The parasitoids used in this bioassay were not isolated to assess their cause of death because they were not in contact with the fungi.

In both bioassays, the following biological parameters were assessed daily: (1) number of parasitized *D. fovealis* eggs; (2) percentage of emergence (%E) calculated as %E = (TE/TO) × 100, where TE is the total number of emerged adults and TO is the total number of parasitized eggs; (3) longevity of emerged adults; (4) egg-to-adult development period; (5) offspring sex ratio (R), computed as R = TF/TE, where TF is the total number of emerged females. The percentage reductions (PR) in emergence due to parasitized eggs, adult longevity, and percentage of parasitism relative to the control were calculated using the following equation: PR (%)= [1 − (Q/q) × 100], where PR is the percentage reduction in the relevant biological parameter, Q is the average value of the parameter for the EF treatment, and q represents the mean value of the parameter obtained in the control.

The selectivity/toxicity of *B. bassiana* isolates and commercial bioinsecticides to egg parasitism by *T. atopovirilia* and *T. pretiosum* was classified according to the IOBC (International Organization for Biological Control) criteria for laboratory tests, where: 1 = innocuous (<30%), 2 = slightly harmful (30–79%), 3 = moderately harmful (80–99%), and 4 = harmful (>99%) [32]. The experimental design was completely randomized, with four treatments and one control per parasitoid. Fifteen replicates were used per combination, and each replicate consisted of a tube with *D. fovealis* eggs (a paper strip with 20 eggs) and a parasitoid.

### 2.6. Statistical Analysis

Data were checked for normality and homoscedasticity using the Shapiro-Wilk and Bartlett tests, respectively [33,34]. When required, data were arcsine transformed. Data from the free-choice test were analyzed using the Wilcoxon non-parametric test. In the pre- and post-parasitism bioassays, data were submitted to an analysis of variance (ANOVA), with period of parasitism, *Trichogramma* species, and entomopathogen treatment, as well as their interactions, as fixed factors. Means were compared with the Tukey honest significant difference (HSD) test [35]. Statistical analyses were conducted in R v.3.6.2 [36].

## 3. Results

### 3.1. Trichogramma atopovirilia and Trichogramma pretiosum Parasitism in Free-Choice Test

**Figure 1:**
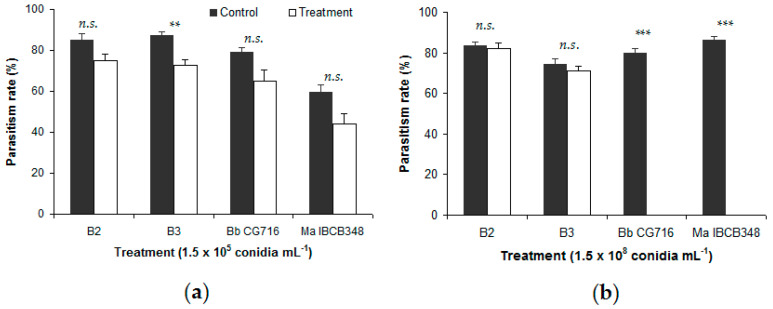
Percentage of *Duponchelia fovealis* eggs parasitized by *Trichogramma atopovirilia* in free-choice tests, comparing eggs sprayed with *Beauveria bassiana* strains (B2 and B3) and commercial bioinsecticides (Bb CG716 and Ma IBCB348) vs. control solutions: (**a**) 1.5 × 10^5^ conidia mL^−1^; (**b**) 1.5 × 10^8^ conidia mL^−1^. Error bars indicate standard errors (n = 15). Significant differences between a given treatment (entomopathogen) and its corresponding control (water), according to the Wilcoxon test, are shown over the bars as follows: n.s. = not significant; ** = *p*-value < 0.01; *** = *p*-value < 0.001.

**Figure 2:**
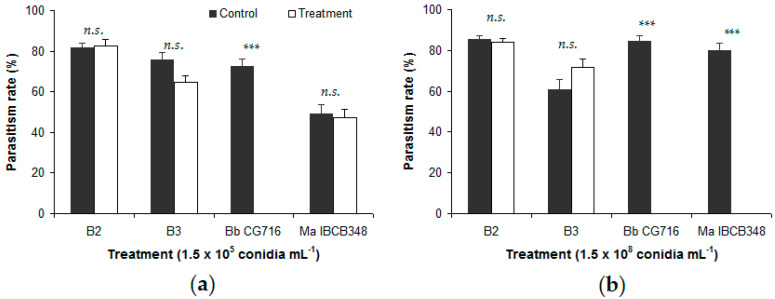
Percentage of *Duponchelia fovealis* eggs parasitized by *Trichogramma pretiosum* in free-choice tests, comparing eggs sprayed with *Beauveria bassiana* strains (B2 and B3) and commercial bioinsecticides (Bb CG716 and Ma IBCB348) vs. control solutions: (**a**) 1.5 × 10^5^ conidia mL^−1^; (**b**) 1.5 × 10^8^ conidia mL^−1^. Error bars indicate standard errors (n = 15). Significant differences between a given treatment (entomopathogen) and its corresponding control (water), according to the Wilcoxon test, are shown over the bars as follows: n.s. = not significant; *** = *p*-value < 0.001.

In general, the B2 and B3 strains did not affect the percentage of *D. fovealis* eggs parasitized by either *Trichogramma* species (Figure 1 and Figure 2).

No significant differences were detected between either *B. bassiana* strain and the respective control in the percentage of eggs parasitized, except for B3 at 1.5 × 10^5^ conidia mL^−1^ in the presence of *T. atopovirilia* (Figure 1a). In contrast, commercial bioinsecticides affected the parasitism process negatively. Eggs sprayed with 1.5 × 10^8^ conidia mL^−1^ of Bb CG716 or Ma IBCB348 were not parasitized by either *Trichogramma* species, whereas their controls showed parasitism rates of between 79.9% and 86.2% of eggs (Figure 1b and Figure 2b). Eggs sprayed with Bb CG716 at the lowest concentration (1.5 × 10^5^ conidia mL^−1^) were not parasitized by *T. pretiosum* (Figure 2a), while parasitism by *T. atopovirilia* remained unaffected (Figure 1a). Spraying with Ma IBCB348 at 1.5 × 10^5^ conidia mL^−1^ did not affect the parasitism behavior of either *Trichogramma* species (Figure 1a and Figure 2a).

Infection rates among parasitoids kept under controlled conditions for 14 days were uniformly low (<30%) following exposure to both *B. bassiana* strains and commercial bioinsecticides at concentrations of 1.5 × 10^5^ conidia mL^−1^ (F_3119_ = 1.22; *p*-value = 0.305) or 1.5 × 10^8^ conidia mL^−1^ (F_3119_ = 0.97; *p*-value = 0.408). Moreover, no differential tolerance to EF products was observed between *T. atopovirilia* and *T. pretiosum* at both 1.5 × 10^5^ conidia mL^−1^ (F_1119_ = 0.33; *p*-value = 0.565) and 1.5 × 10^8^ conidia mL^−1^ (F_1119_ = 0.07; *p*-value = 0.795). Mortality in the control groups was not fungus-related.

### 3.2. Spraying of Entomopathogenic Fungi Pre- and Post-Parasitism (No-Choice Tests)

The biological parameters of the *Trichogramma* species (parasitism rate, emergence rate, offspring sex ratio, and duration of the egg-to-adult period) were significantly affected by the period in which parasitoids were exposed to the EF (pre- or post-parasitism), the EF treatments themselves and the interactions among them, as shown by a full ANOVA model (*p*-values ranging from < 0.001 to 0.039). Moreover, sex ratio (*p*-value < 0.001) and duration of the egg-to-adult period (*p*-value = 0.013) differed between *Trichogramma* species. Consequently, data were analyzed separately for each period of parasitism and *Trichogramma* species.

**Figure 3:**
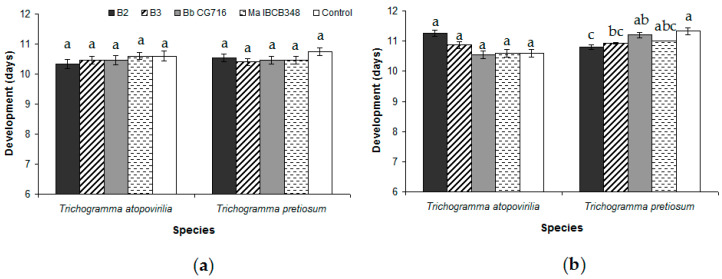
Average length of the egg-to-adult period of development of *Trichogramma atopovirilia* and *Trichogramma pretiosum* individuals emerged from *Duponchelia fovealis* eggs sprayed with *Beauveria bassiana* strains (B2 and B3) and commercial bioinsecticides (Bb CG716 and Ma IBCB348) pre- (**a**) and post-parasitism (**b**). Error bars are standard errors (n = 15). Small letters over the bars indicate significant differences among treatments according to the Tukey HSD test (*p*-value < 0.05).

**Figure 4:**
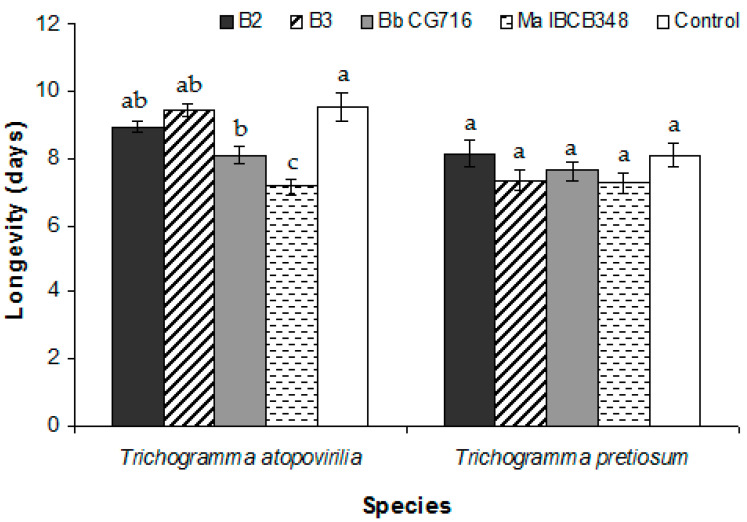
Average longevity of *Trichogramma atopovirilia* and *Trichogramma pretiosum* individuals emerged from *Duponchelia fovealis* eggs sprayed with *Beauveria bassiana* strains (B2 and B3) and commercial bioinsecticides (Bb CG716 and Ma IBCB348) pre-parasitism. Error bars are standard errors (n = 15). Small letters over the bars indicate significant differences among treatments according to the Tukey HSD test (*p*-value < 0.05).

EF application during the pre-parasitism period did not result in any change in the biological parameters studied relative to the control treatment (Table 1). In the case of *T. pretiosum*, the only significant difference was observed in the emergence rate between the B2 and B3 treatments (Table 1). In addition, the percentage of emerged adults was lower in *T. atopovirilia* than in *T. pretiosum* when they were treated with B2, though the opposite was observed following application of Bb CG716 (Table 1). The length of the egg-to-adult period was not affected by any treatment in either of the *Trichogramma* species (Figure 3a). The longevity of *T. atopovirilia* was reduced by the application of Bb CG716 and Ma IBCB348, while the *B. bassiana* strains had no effect relative to the control (Figure 4). The treatments did not affect the longevity of *T. pretiosum* (Figure 4).

The percentage of eggs parasitized in the post-parasitism bioassay was not affected by the spraying of EF for either *Trichogramma* species (Table 2). The percentage of emerged *T. atopovirilia* adults following B2 application was lower than the control (Table 2). The offspring sex ratio in *T. atopovirilia* was not affected by the entomopathogens; on the other hand, for *T. pretiosum*, this biological trait was reduced by all the entomopathogen treatments considered in this study, especially B2 (Table 2). The only parameter that was significantly different between the *Trichogramma* species was the offspring sex ratio, which was greater for *T. pretiosum* under the control, Bb CG716 and Ma IBCB348 treatments, and greater for *T. atopovirilia* under the B2 treatment (Table 2). The length of the egg-to-adult period was not affected by EF in *T. atopovirilia*, while *T. pretiosum* individuals had a shorter development under the B2 and B3 treatments than under the control treatment (Figure 3b). The *B. bassiana* strains and bioinsecticides considered in this study caused reductions of under 30% in the percentage of eggs parasitized, emergence of adults, and longevity in both the pre- and post-parasitism bioassays for the two *Trichogramma* species. Therefore, all EF treatments were classified as innocuous (Class 1) according to the IOBC criteria.

## 4. Discussion

In the free-choice bioassay, parasitism rate was completely absent in the treatments with commercial bioinsecticides at 1.5 × 10^8^ conidia mL^−1^; however, at a lower concentration (1.5 × 10^5^ conidia mL^−1^), Bb CG716 did not affect the parasitism rate of *T. atopovirilia*, although it completely inhibited parasitism of *T. pretiosum*. In contrast, the *B. bassiana* strains tested (B2 and B3) did not affect the parasitism rates of either *Trichogramma* species. In a similar study, significant reductions in the parasitism rate of *T. pretiosum* were observed in a free-choice test when *B. bassiana* strains Unioeste 47 and Unioeste 57 were applied at concentrations of 1.5 × 10^9^ conidia mL^−1^ on the eggs of *Ephestia kuehniella* Zeller (Lepidoptera: Pyralidae) [22]. These contrasting observations may be due to differences in the conidia concentrations applied, the *B. bassiana* strains used, or the target organism [22]. It is possible that *T. pretiosum* females can recognize the presence of a harmful or repellent substance deposited on the host eggs before parasitizing them [22,37]. Females may be able to recognize whether the host is infected by a fungus by walking back and forth over the eggs, touching them with the gustatory sensilla of their antennae [22,37,38]. Meanwhile, commercial products may contain carrying substances in their formulations, which could partially explain the stronger effect of these bioinsecticides on the *Trichogramma* species studied.

The pre- and post-parasitism (no-choice) EF application tests showed that all of the products were innocuous according to the IOBC selectivity/toxicity classification since parasitism was reduced by <30%. These results are consistent with previous observations on *T. pretiosum* [22]. Similarly, prior studies did not observe significant reductions in parasitism by *T. pretiosum* and *T. atopovirilia* when *B. bassiana* and *M. anisopliae* strains were applied on host eggs in no-choice tests [23,27,39]. The pattern observed in the current study of parasitoids being repelled by EF in the free-choice tests but accepting the infected eggs in the no-choice tests may indicate that the host eggs are suitable for parasitism by *T. atopovirilia* and *T. pretiosum*. In fact, *Trichogramma* females probe potential host eggs prior to parasitizing them by inserting the ovipositor and verifying the nutritional quality of the egg [40].

The egg-to-adult development period was not affected in either of the *Trichogramma* species by any entomopathogen treatment in the no-choice tests performed prior to parasitism in this study. In contrast, a minimal effect was observed in *T. pretiosum* development following post-parasitism with EF application. This is consistent with previous studies of *T. pretiosum* in which host eggs were sprayed with strains of *B. bassiana* and *M. anisopliae* [22,27]. Alterations of the length of the *Trichogramma* parasitoids’ egg-to-adult period may be related to changes in the host caused by the fungus [38]. However, the near absence of alterations in the egg-to-adult period reinforces prior test findings that *D. fovealis* eggs were not affected by EF [21].

The percentage of emergence in the no-choice tests performed in this study was not significantly affected by most entomopathogen treatments, while any significant reductions were under 30% relative to the control. Of all the pre-parasitism treatments, only the *B. bassiana* strains B2 and B3 caused reductions in this biological parameter. Meanwhile, only *T. atopovirilia* had a significantly lower percentage of emerged adults following post-parasitism application of B2 (75.8%) compared with the control (93.5%). These results are consistent with previous research showing that the emergence of *T. pretiosum* adults did not differ following pre- and post-parasitism spraying with *B. bassiana* strains (no-choice test) [22]. In contrast, significant reductions of *T. pretiosum* emergence have been reported as a result of the application of *M. anisopliae* (Unioeste 22) to eggs of *E. kuehniella* in no-choice tests, though this reduction was only observed following pre-parasitism application and accounted for less than 30% [27]. Furthermore, these authors did not observe significant reductions in this parameter when eggs were sprayed with *B. bassiana* strain Unioeste 1 [27]. Moreover, a study found that *B. bassiana* (Boveril WP PL63) and *M. anisopliae* (Metarril WP E9) sprayed onto eggs of *Spodoptera frugiperda* (Smith) (Lepidoptera: Noctuidae) prior to parasitism did not affect the emergence rate of *T. atopovirilia* [39].

The offspring sex ratio differed between the two *Trichogramma* species considered in this study. In both cases, this ratio was affected by EF application during the post-parasitism period. These results differ from previous observations of *T. pretiosum* in which offspring sex ratio was not altered by the host eggs being sprayed with *B. bassiana* and *M. anisopliae* strains either pre- or post- parasitism [22,27]. Alterations in sex ratio could occur due to interference with the behavior of *Trichogramma* adults or an effect on the fertilization of ova [41]. However, although significant changes were observed in the offspring sex ratio for both *Trichogramma* species, this ratio was greater than 0.6 in all treatments, which is considered satisfactory since sex ratio values of over 0.5 are suitable for mass rearing and do not hinder the success of biological control programs [42].

Concerning the longevity of emerged adults, only the commercial biopesticides significantly affected this trait in *T. atopovirilia*, in which longevity was reduced following pre-parasitism application of these compounds. In contrast, previous research did not report changes in longevity due to pre-parasitism application of *B. bassiana* and *M. anisopliae* strains (no-choice tests) [39]. The longevity of *T. pretiosum* was not affected by any of the treatments, which is consistent with previous reports involving *T. pretiosum* and *B. bassiana* and *M. anisopliae* strains [22,27].

The percentage of fungus-related mortality of adult parasitoids in the current study was low and consistent across the strains applied, which is crucial for the viability of combining parasitoids and EF within IPM strategies. The mortality patterns may be explained by the way in which EF were applied in the current study (no direct contact between EF and parasitoids) and the period of application. Previous research has shown that confirmed fungus-related mortality depends on the virulence of fungal strains, EF species, *Trichogramma* species, and the application period (pre- or post-parasitism) [22,27,39]. Furthermore, the interactions between the EF and *Trichogramma* species were minimal for all biological parameters considered in this study, especially when compared with chemical insecticides [11,43,44,45].

The effectiveness of the commercial biopesticides and EF strains tested in the present study in controlling *D. fovealis* has already been demonstrated [20]. Meanwhile, this study suggests that the residual effects of these biocontrol agents on *T. atopovirilia* and *T. pretiosum* were minimal. Moreover, these EF strains have shown low adverse impacts on two natural enemies of *D. fovealis* that are commonly present in Brazilian strawberry fields, *Podisus nigrispinus* (Dallas) (Hemiptera: Pentatomidae) and *Harmonia axyridis* (Pallas) (Coleoptera: Coccinellidae) [16]. Thus, the combined use of these biological control agents may be suitable for IPM programs. The use of EF with a parasitoid may improve the effectiveness of biological control strategies since these organisms act on the same pest species at different stages of its life cycle [23,39].

Overall, the results of this study suggest that the safety of EF is specific and depends on the fungal strain and conidia concentration, as well as the particular host and parasitoid under evaluation. Free and no-choice bioassays are, therefore, essential to confirm the selectivity and to better understand the effect that a given EF strain might have on a certain species of the *Trichogramma* genus. Because the results of free and no-choice bioassays can vary greatly, performing a single test would lead to biases or partial conclusions on the selectivity of an EF strain for a given *Trichogramma* species [22,23]. Moreover, combining the two approaches allows for a better understanding of the situations in which the EF would cause lower impacts on the performance and biological parameters of these parasitoids, which can inform the design of IPM strategies.

## 5. Conclusions

This study demonstrates that the adverse effects observed on the biological parameters of *T. pretiosum* and *T. atopovirilia* were minimal, suggesting that the *B. bassiana* strains and the commercial products tested could be harmless to these parasitoids; however, further research is needed to confirm this. Effectiveness against a particular pest and the innocuity against non-target organisms are key characteristics of good IPM practices. Thus, the results obtained constitute a first step in designing effective *D. fovealis* control strategies involving both EF and parasitoids. Future research should investigate the sub-lethal effects of these fungal strains on such parasitoids, besides involving field studies.

## Figures and Tables

**Table 1:** Biological parameters (% eggs parasitized, % emerged adults, and offspring sex ratio) of *Trichogramma atopovirilia* and *Trichogramma pretiosum* individuals emerged from *Duponchelia fovealis* eggs sprayed with *Beauveria bassiana* strains (B2 and B3) and commercial bioinsecticides (Bb CG716 and Ma IBCB348) pre-parasitism. Data represent averages ± standard errors (n = 15).

Species	Control	B2	B3	Bb CG716	Ma IBCB348
% eggs parasitized					
*T. atopovirilia*	87.2 ± 2.7 AB ^1^	89.5 ± 2.6 A	82.3 ± 2.9 AB	78.4 ± 2.2 B	78.3 ± 3.3 AB
*T. pretiosum*	70.1 ± 4.1 B	86.8 ± 3.6 AB	82.9 ± 3.8 AB	82.1 ± 2.3 AB	85.7 ± 1.9 A
Significance	**	n.s.	n.s.	n.s.	n.s.
% emerged adults					
*T. atopovirilia*	93.3 ± 2.6 AB	85.7 ± 2.5 B	87.1 ± 2.1 B	96.1 ± 1.6 A	91.5 ± 2.1 AB
*T. pretiosum*	92.3 ± 2.4 AB	97.3 ± 1.0 A	87.1 ± 2.0 B	87.7 ± 2.6 AB	89.0 ± 2.4 AB
Significance	n.s.	**	n.s.	*	n.s.
Sex ratio					
*T. atopovirilia*	0.72 ± 0.02 A	0.72 ± 0.01 A	0.72 ± 0.02 A	0.63 ± 0.03 A	0.70 ± 0.02 A
*T. pretiosum*	0.71 ± 0.03 A	0.69 ± 0.02 A	0.77 ± 0.01 A	0.73 ± 0.01 A	0.74 ± 0.02 A
Significance	n.s.	n.s.	n.s.	n.s.	n.s.

^1^ Capital letters in the row indicate significant differences among entomopathogen treatments for a given *Trichogramma* species according to the Tukey HSD test at *p* < 0.05. The significant differences between the response of the two *Trichogramma* species under a given entomopathogen treatment detected by ANOVA are reported as follows: n.s. = not significant; * *p* < 0.05; ** *p* < 0.01.

**Table 2:** Biological parameters (% eggs parasitized, % emerged adults and offspring sex ratio) of *Trichogramma atopovirilia* and *Trichogramma pretiosum* individuals emerged from *Duponchelia fovealis* eggs sprayed with *Beauveria bassiana* strains (B2 and B3) and commercial bioinsecticides (Bb CG716 and Ma IBCB348) post-parasitism. Data represent averages ± standard errors (n = 15).

Species	Control	B2	B3	Bb CG716	Ma IBCB348
% eggs parasitized					
*T. atopovirilia*	89.8 ± 1.1 A ^1^	81.3 ± 1.9 A	83.3 ± 2.2 A	90.2 ± 2.1 A	85.1 ± 3.5 A
*T. pretiosum*	80.7 ± 4.3 A	90.9 ± 1.8 A	84.4 ± 2.6 A	88.2 ± 2.3 A	80.8 ± 3.2 A
Significance	n.s.	n.s.	n.s.	n.s.	n.s.
% emerged adults					
*T. atopovirilia*	93.5 ± 1.8 A	75.8 ± 1.7 B	91.4 ± 2.4 A	93.1 ± 2.0 A	92.7 ± 2.2 A
*T. pretiosum*	93.3 ± 2.3 A	91.8 ± 1.9 A	93.7 ± 2.3 A	95.6 ± 2.2 A	93.3 ± 1.7 A
Significance	n.s.	n.s.	n.s.	n.s.	n.s.
Sex ratio					
*T. atopovirilia*	0.66 ± 0.02 A	0.72 ± 0.01 A	0.65 ± 0.01 A	0.70 ± 0.01 A	0.69 ± 0.03 A
*T. pretiosum*	0.95 ± 0.02 A	0.66 ± 0.01 C	0.70 ± 0.02 BC	0.77 ± 0.03 BC	0.80 ± 0.03 B
Significance	***	**	n.s.	*	*

^1^ Capital letters in the row indicate significant differences entomopathogen treatments for a given *Trichogramma* species according to the Tukey HSD test at *p* < 0.05. The significant differences between the response of the two *Trichogramma* species under a given entomopathogen treatment detected by ANOVA are reported as follows: n.s. = not significant; * *p* < 0.05; ** *p* < 0.01; *** *p* < 0.001.
